# Genetic Screens for Mutations Affecting Development of Xenopus tropicalis

**DOI:** 10.1371/journal.pgen.0020091

**Published:** 2006-06-09

**Authors:** Tadahiro Goda, Anita Abu-Daya, Samantha Carruthers, Matthew D Clark, Derek L Stemple, Lyle B Zimmerman

**Affiliations:** 1 Division of Developmental Biology, National Institute for Medical Research, The Ridgeway, Mill Hill, London, United Kingdom; 2 Vertebrate Development and Genetics, Wellcome Trust Sanger Institute, Wellcome Trust Genome Campus, Hinxton, Cambridge, United Kingdom; University of Pennsylvania School of Medicine, United States of America

## Abstract

We present here the results of forward and reverse genetic screens for chemically-induced mutations in *Xenopus tropicalis.* In our forward genetic screen, we have uncovered 77 candidate phenotypes in diverse organogenesis and differentiation processes. Using a gynogenetic screen design, which minimizes time and husbandry space expenditures, we find that if a phenotype is detected in the gynogenetic F2 of a given F1 female twice, it is highly likely to be a heritable abnormality (29/29 cases). We have also demonstrated the feasibility of reverse genetic approaches for obtaining carriers of mutations in specific genes, and have directly determined an induced mutation rate by sequencing specific exons from a mutagenized population. The *Xenopus* system, with its well-understood embryology, fate map, and gain-of-function approaches, can now be coupled with efficient loss-of-function genetic strategies for vertebrate functional genomics and developmental genetics.

## Synopsis

Amphibian embryos can be used to understand how all vertebrates, including mammals, develop from fertilized single-celled eggs to establish a body plan and form different cell types and functional organs. Genetic methods are used to analyze what goes wrong in embryos lacking working versions of individual genes, and help to understand those genes' specific functions. However, genetic analysis of previously studied amphibians has been difficult because of these species' long generation time and complex genetic structure. The authors have established methods for systematically studying disrupted genes in the frog *Xenopus tropicalis,* which has a relatively short generation time, simple genetic structure, and an easily studied externally-developing embryo. They describe their methods for creating and characterizing X. tropicalis mutations, using both forward genetics (where a mutation's effects on the embryo are first characterized, then the DNA defect is later identified) and reverse genetics (where animals carrying mutations in a known DNA sequence are first identified, and the effects of that mutation are characterized subsequently). Studies of amphibian development using tissue culture, transplantation, and molecular tools have been fundamental to understanding vertebrate early development. These studies will be greatly enriched by the addition of forward and reverse genetics to complement emerging genomic tools.

## Introduction

Genetic studies have arguably contributed more to our understanding of animal development than any other approach. Invertebrate genetic models have helped identify the transcriptional control networks underpinning the basic animal body plan [1,2]; among vertebrates, the mouse has been an especially powerful tool for genetic studies since the development of gene targeting [3,4], but forward screens for embryonic mutations in this system are challenging due to the intrauterine mode of development. Zebrafish screens have benefited from its high fecundity, short generation time, and rapid development of externally fertilized, transparent embryos, resulting in the identification of a large number of genes controlling developmental processes [5–8], and reverse genetic resources are becoming available [9,10]. An ancestral teleost genome duplication, and subsequent partitioning of gene subfunctions, permits mutational analysis of paralog roles, which may be obscured by pleiotropic effects of orthologs with simpler evolutionary histories. However, where duplicated genes have not diverged functionally, they may be inaccessible to forward genetic screens. While it is not clear whether an increased redundancy has been retained relative to other vertebrates, subfunctionalization and neofunctionalization in teleosts have resulted in a significant degree of reorganization of genetic roles [11]. Since teleosts are also the most evolutionarily diverse vertebrates, systematic comparison with canonical tetrapod genomes is essential for understanding gene function in vertebrate development.

The amphibian embryo, with its well-characterized embryology, fate map, and amenability to a variety of gain-of-function techniques, is an alternative tetrapod vertebrate substrate for genetic screens. However, the allotetraploid origin and long generation time of the most intensively studied amphibian, *Xenopus laevis,* reduce its utility in this approach. A related pipid frog, *X. tropicalis,* has been adopted for the same suite of embryological, molecular, and transgenic approaches as *X. laevis,* but is a true diploid with a genome size (ten chromosomes, 1.7 × 10^9^ bp) approximately half that of *X. laevis,* and which reaches sexual maturity in as little as 3 mo [12,13]. Large-scale multigeneration husbandry is also facilitated by its small size, with a volume ~1/8 that of *X. laevis.* Genomics support for X. tropicalis research comprises over 1,000,000 EST sequences (http://www.ncbi.nlm.nih.gov/dbEST/dbEST_summary.html), including an annotated set of full-length cDNAs (http://www.sanger.ac.uk/Projects/X_tropicalis/X_tropicalis_cDNA_project.html), BAC libraries (http://bacpac.chori.org/libraries.php), a genome sequence assembly approaching 8× coverage (http://genome.jgi-psf.org/Xentr4/Xentr4.home.html), plus an increasingly dense meiotic map based on simple sequence repeat (SSR) markers currently comprising 11 linkage groups (http://tropmap.biology.uh.edu/map.html). The X. tropicalis system thus offers a unique opportunity to combine forward and reverse genetic and genomic approaches with classical embryological, molecular, and gain-of-function analytical techniques in a single model vertebrate embryo [13–16].

In this pilot study, we have pursued a strategy of in vitro chemical mutagenesis of mature X. tropicalis sperm followed by in vitro fertilization, maturation of an F1 generation, and both forward screens of gynogenetic F2 embryos and reverse genetic approaches. Chemical mutagenesis permits more efficient induction of mutations than extant insertional strategies [17,18], and the resulting phenotypes are more likely to be associated with single gene defects than those produced by γ-radiation–induced large deletions [19]. Gynogenetic F2 embryos derived from F1 candidate carriers can reveal recessive phenotypes with only one generation intervening between mutagenesis and screening, greatly reducing husbandry and time requirements for our screen. This method has previously been used to identify naturally occurring mutations in X. laevis [20] and X. tropicalis [21] as well as induced mutations in zebrafish [22–24]. We further streamlined our screen by only scoring postneurulation developmental events, both in order to avoid background noise associated with epigenetic gastrulation defects and because early developmental processes are often accessible to zygotic loss-of-function studies with knockdown approaches.

In a complementary reverse genetic approach, we have employed a TILLING strategy (targeting induced local lesions in genomes) [10,25,26,48] to obtain mutations in known sequences. Direct sequencing of genomic PCR products from a library of F2 frogs is used to identify carriers, which are then recovered from a cognate library of frozen F1 sperm for phenotypic analysis. In addition, sequence analysis of a number of exons from a large number of individual mutation-carrying F2 embryos has been used to calculate an induced mutation rate.

## Results

### A Pilot Gynogenetic Screen

**Figure 1:**
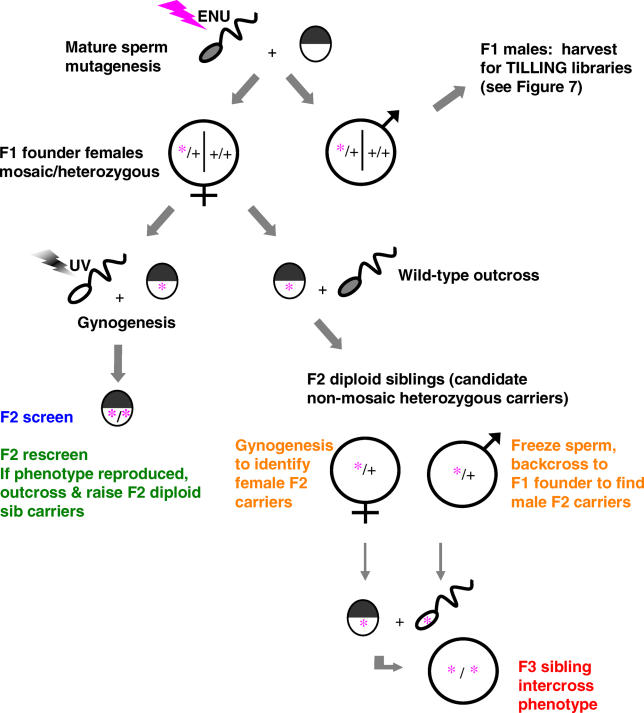
Forward Screen Strategy Following ENU mutagenesis of postmeiotic sperm and fertilization of wild-type eggs, a founder F1 generation was raised. Males were used in a reverse genetic strategy (see Figure 7). F1 females were used to generate gynogenetic embryos that were screened for embryonic defects. F1 females carrying defects were outcrossed and the resulting F2 embryos screened for carriers, then sibling intercrossed. Color code indicates status of specific mutations (see Figure 2 and Tables 2 and 3): red for phenotypes confirmed in the progeny of a conventional F2 sibling intercross, orange for phenotypes confirmed heritable by backcross or F2 gynogenesis, green for phenotypes observed twice from gynogenesis of an individual F1 female, and blue for phenotypes observed once and not yet retested.

To minimize time and colony space requirements, we implemented a two-generation gynogenetic screen design (Figure 1). In this approach, recessive mutations can be identified in the progeny of individual heterozygous females (F1 founders) derived from in vitro mutagenized sperm (G0). By generating haploid F2 embryos and treating them with pressure or coldshock to suppress polar body formation, it is possible to rescue a diploid state on which recessive phenotypes can be detected [20–24]. Mendelian phenotypic ratios will not generally be recovered with this approach. Egg and polar body chromosomal complements are the products of meiotic recombination; thus, distal loci in gynogenetic embryos are more likely to be derived from nonsister chromatids and hence heterozygous, resulting in a higher frequency of recovery of centromerically linked recessive mutations [5,20,27,28]. Since we have also employed in vitro mutagenesis, mosaicism in the F1 generation already precludes production of predictable Mendelian phenotypic ratios in conventional sibling crosses. Conventional Mendelian genotypic ratios can be obtained in sibling crosses following the identification of carriers in nonmosaic F2 and subsequent generations.

**Table 1:**
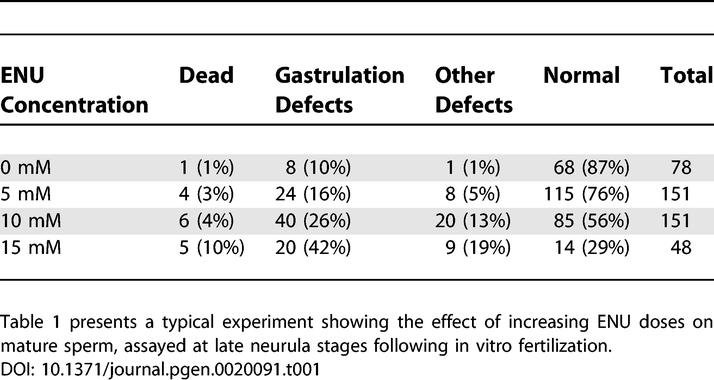
ENU Dose–Response

**Figure 2:**
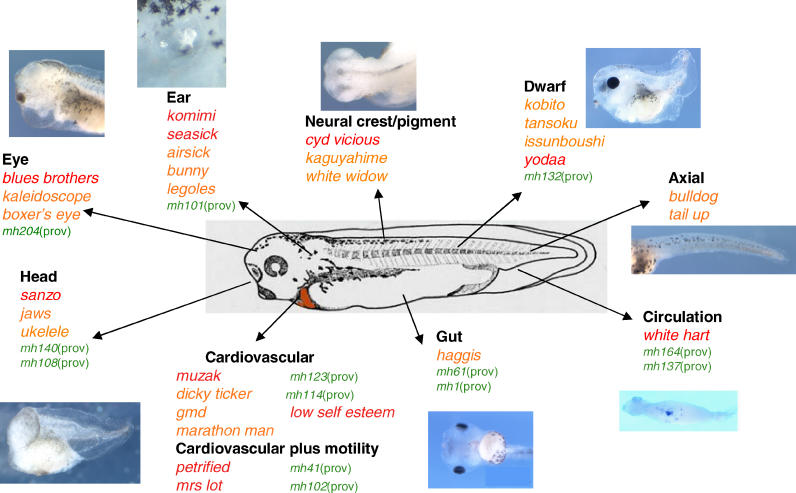
Phenotypes Detected Defects were sorted into ten broad categories (shown with representative images): eye *(zatoichi),* inner ear and otolith *(komimi),* neural crest/pigment *(cyd vicious),* dwarf *(issunboushi),* axial *(bulldog),* circulation *(desert tad),* gut *(haggis),* cardiovascular system and motility, and head *(troll)*. Color code (green, orange, red) is described in Figure 1 and used in Tables 2 and 3 to denote current confirmation status of individual mutations in the pipeline. Provisional alleles (“prov,” green) have not yet been assayed for heritability.

**Table 2:**
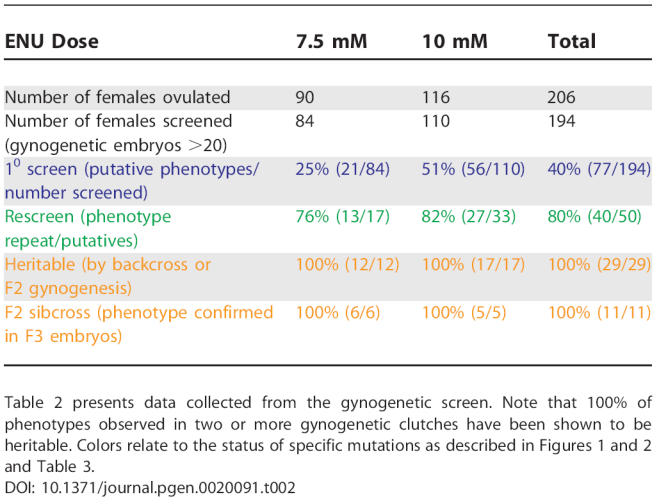
Forward Screen Statistics

**Table 3:**
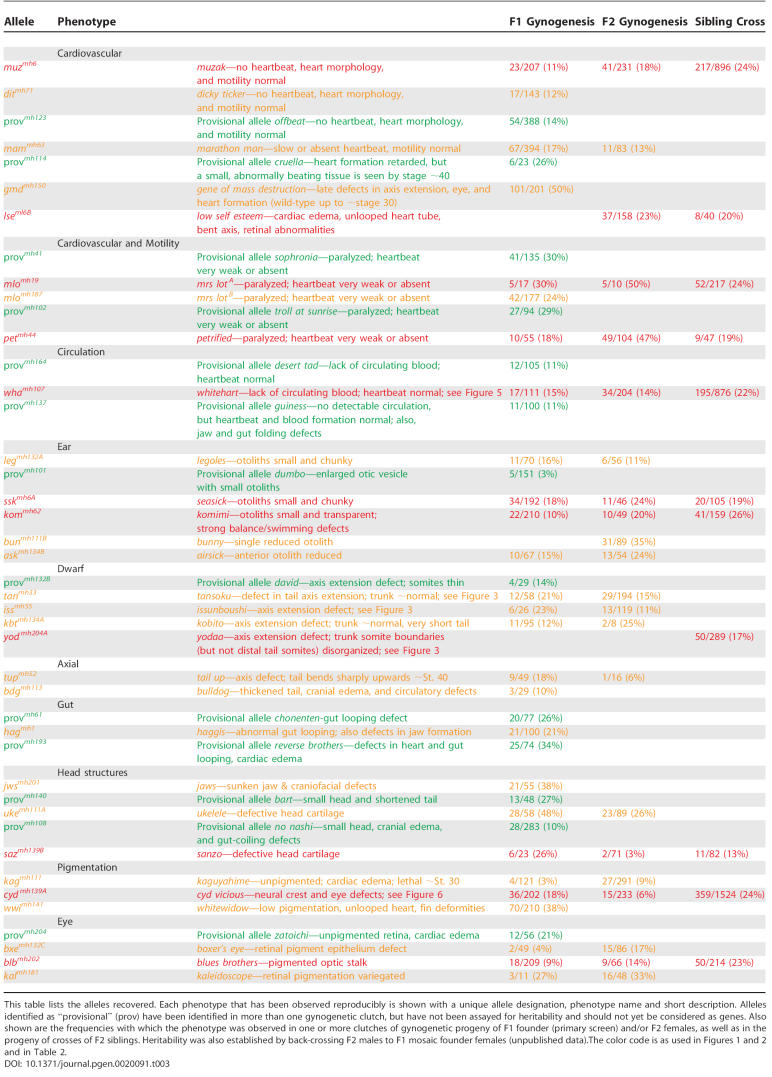
Alleles Recovered

**Figure 3:**
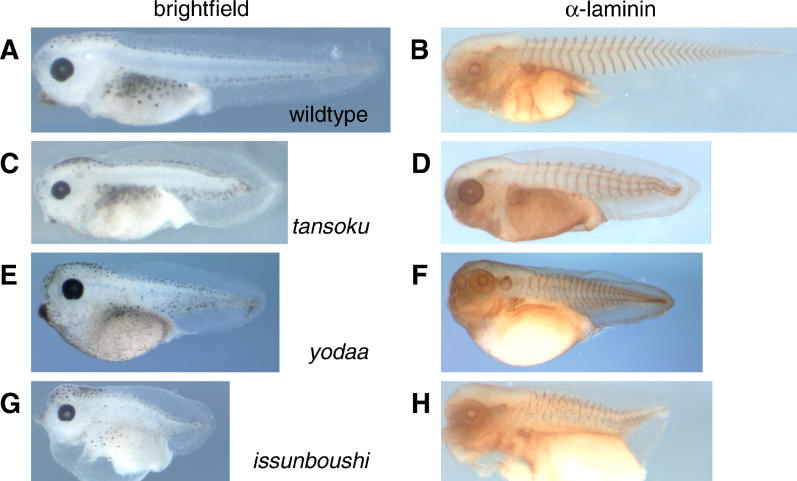
Axis Extension Mutations The dwarf phenotypes *tansoku*, *yodaa*, and *issunboushi* show relatively normal head and trunk structures, but are defective in tail extension. Anti-laminin immunohistochemistry reveals discrete defects in axial structures, with *tansoku* ([C] and [D]) displaying a reduced number of relatively well-ordered somites, *issunboushi* ([G] and [H]) showing highly disordered intersomitic boundaries, and *yodaa* ([E] and [F]) displaying an intermediate phenotype.

**Figure 4:**
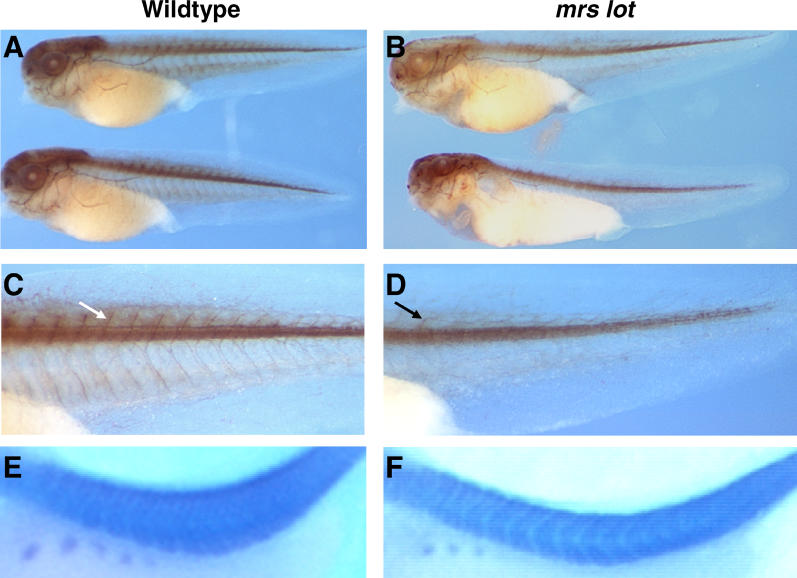
The *mlo* Mutation Exhibits Paralysis and Motor Neuron Defects Neural tissue of *mlo* and diploid control was stained with the HNK-1 antibody. In wild-type tadpoles ([A] and [C]), motor neuron axons (white arrow) travel down the intermyotomal cleft from the neural tube; in *mlo* ([B] and [D]) the axonal tracts (black arrow) wander away from the intermyotomal space. In situ hybridization with cardiac actin ([E] and [F]) shows that somite structure is relatively unaffected.

**Table 4:**
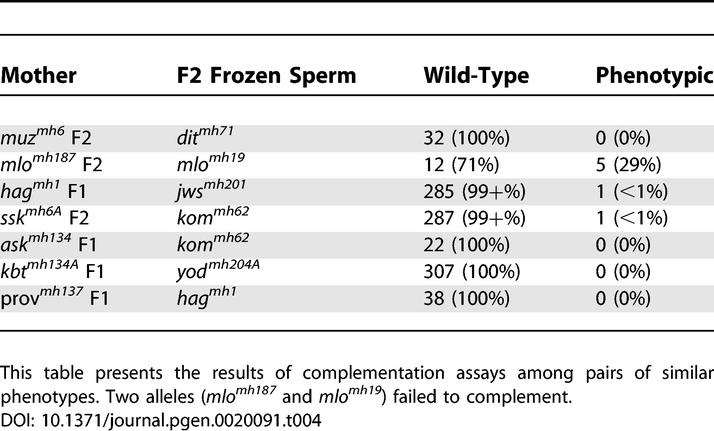
Complementation Analysis

**Figure 5:**
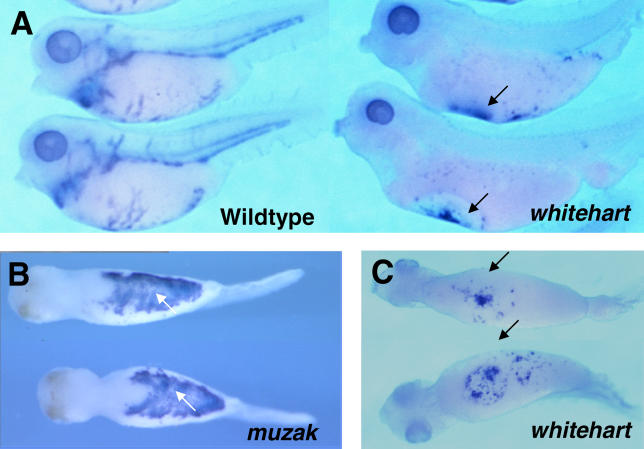
*wha* Embryos Show Defects in Hematopoeisis Whole mount in situ hybridization with α-globin suggests that *wha* blood distribution is aberrant ([A] and [C]), with reduced globin staining pooled ventrally (black arrows) rather than distributed throughout the circulatory system as in wild-type tadpoles. Comparison of ventral views of *wha* (C) globin staining with that of *muzak* (B), a mutant which is impaired in heart function but not hematopoiesis, leading to ventral pooling of normal levels of blood (white arrows), confirms that *wha* is quantitatively defective in blood formation rather than circulation. See also Tables 6 and 7 for microarray analysis of *wha.*

**Table 5:**
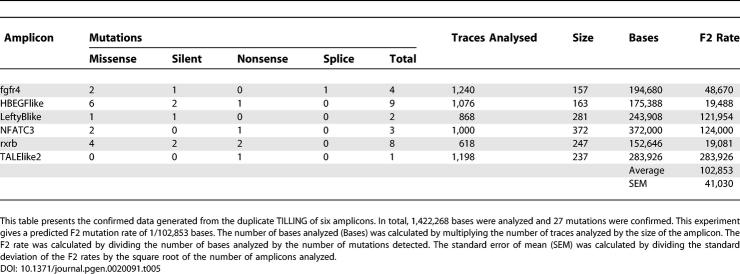
Reverse Screen Statistics

**Figure 6:**
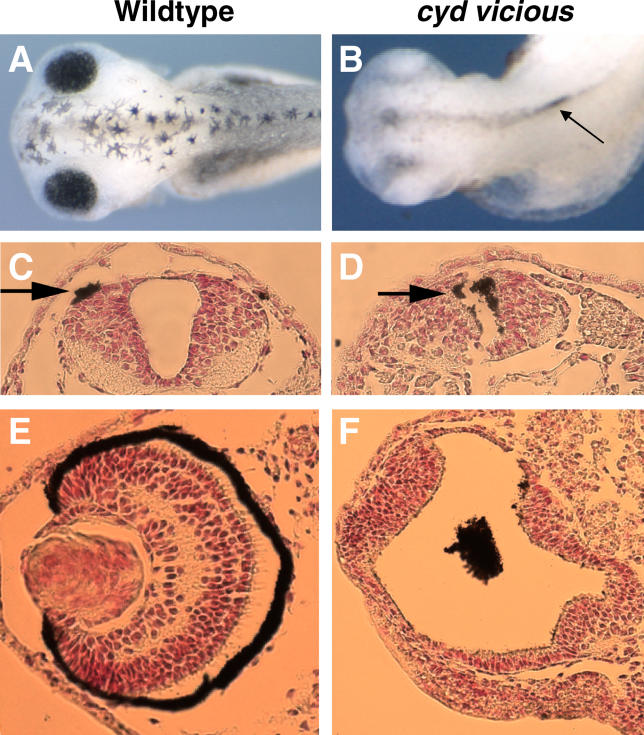
The Mutation *cyd vicious* Displays Neural Crest and Eye Defects Brightfield images of stage ~38 outcrossed sibling wild-type embryo (A) and gynogenetic *cyd* embryo (B). Likely neural crest–derived pigmented cells (arrows, [C] and [D]) fail to migrate in *cyd*, and instead populate the lumen of the neural tube. St. 40 wild-type eye (E) displays laminar organization surrounded by prominent pigmented epithelium. *cyd* eyes form a poorly laminated ball of neural retina surrounding a central mass of pigmented tissue, and no lens tissue is visible (F).

**Table 6:**
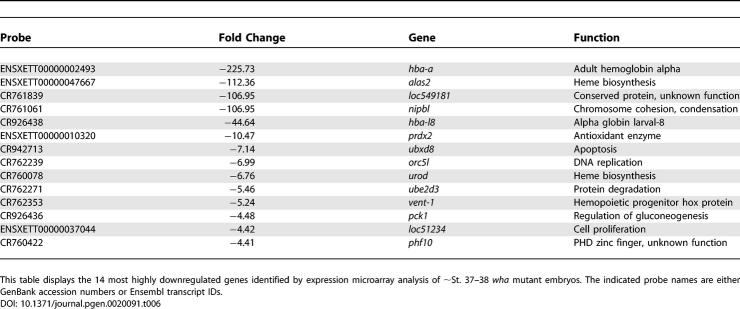
Genes Downregulated in *wha* Mutants

**Figure 7:**
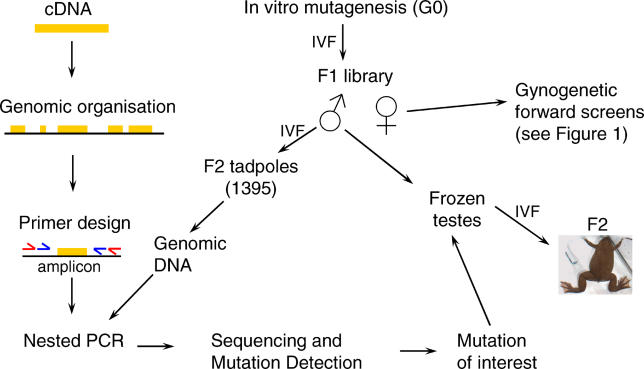
Reverse Screen Strategy ENU-treated sperm (G0) was used to fertilize wild-type eggs (in vitro fertilization), and the resulting F1 families raised to adulthood. F1 males were killed and their testes dissociated, with a portion used to generate F2 tadpoles and the remainder frozen in several aliquots per individual (F1 library). F1 females were used in the gynogenetic forward screens (see Figure 1). F2 genomic DNA was isolated from the tadpoles for reverse genetic (TILLING) screens. Known genomic sequences were used to design nested PCR primers, and then individual F2 tadpole amplicons were sequenced to detect induced mutations. Mutations are then recovered from frozen testes by in vitro fertilization for subsequent phenotypic analysis.

**Table 7:**
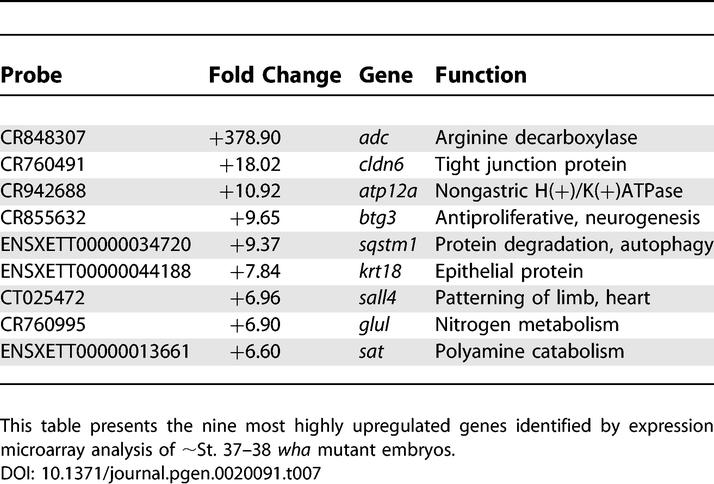
Genes Upregulated in *wha* Mutants

We chose to focus our morphological screen on postneurulation developmental events. Gynogenesis by pressure treatment results in variable recovery of viable embryos from unrelated, nonmutagenized wild-type adults (unpublished data) [21]. The observed range of gastrulation defects are consistent with either partial haploid rescue or endogenous mutations carried by a variety of frog strains [22], creating a noisy background upon which identifying specific mutations is difficult. By discarding embryos that displayed defects prior to neurulation stages, we are able to efficiently screen for later phenotypes in patterning, organogenesis, and differentiation.

The remaining gynogenetic and haploid embryos were examined microscopically and scored for morphological abnormalities and motility defects appearing up to 4 d after fertilization. An F1 female was considered screened when >20 viable gynogenetic F2 neurulae had been examined; this number was chosen as an arbitrary “good average” yield of viable pressure-generated gynognetic neurulae for the purpose of this pilot screen. Females that did not produce embryos with detectable morphological abnormalities were pooled, allowed to recover for >2 mo, and rescreened twice. Females that did produce putative phenotypes were isolated, allowed to recover, and rescreened gynogenetically; F2 outcrossed siblings were then raised from females that reproduced specific gynogenetic phenotypes. Within mature F2 families, female carriers were identified by gynogenesis, and male carriers were identified by backcross to the founding F1 mother. F2 sibling crosses were performed as identified carriers of both sexes became available; where only identified female carriers were available, studies were also performed using gynogenetically produced embryos.

### Mutagen Dosage

In the absence of easily scored homozygous-viable “tester” strains on which to optimize dosage, we sought a relatively high dose that would give substantial dominant effects, but still result in a large number of viable F1 founder embryos. We tested a range of ENU concentrations by treating mature sperm for 1 h, fertilizing wild-type eggs, and scoring the resulting embryos for dominant effects and viability the following day. Table 1 shows a typical experiment, in which the proportion of morphologically abnormal or dead late-neurula-stage embryos increases with ENU concentration over a range from buffer control to 15 mM ENU, consistent with production of dominant and/or synthetic lethal mutations. Choosing doses that were clearly mutagenic, but also provided adequate numbers of viable tadpoles, we generated F1 populations from 7.5 and 10 mM ENU–treated sperm to raise for screening.

### Forward Screen Results

In the gynogenetic progeny of 110 F1 females derived from 10 mM ENU–treated sperm, 56 candidate phenotypes were obtained (Tables 2 and 3). Of these candidate phenotypes, 82% (27/33) were observed again when individual females were rescreened. Moreover, 100% (17/17) of the rescreened phenotypes were identified in a subsequent generation and are therefore heritable. In the 7.5 mM ENU–treated pool, 21 candidate phenotypes were obtained from 84 F1 frogs screened, and 76% (13/17) were confirmed upon rescreening (Table 2). Again, all rescreened phenotypes in the 7.5-mM pool tested (12/12) were found to be heritable. In total, 77 candidate phenotypes from both mutagen doses were isolated, of which 80% (40/50) have been confirmed in a rescreen, and 100% (29/29) of those tested shown to be heritable by backcross or gynogenesis of F2 females. Of the 11 mutations that have been confirmed by conventional crosses of F2 siblings, nine resulted in F3 phenotypic ratios consistent with simple Mendelian inheritance. In seven cases discrete phenotypes were observed to segregate in the gynogenetic or backcross progeny of F2 animals, consistent with the presence of multiple recessive alleles in the F1 founder. These secondary phenotypes may have been masked by the primary phenotype, or they may have occurred at lower frequency in the original screen either due to a higher degree of mosaicism in F1 founders than in subsequent nonmosaic generations or because the responsible locus was further from the centromere and hence uncovered at lower frequency in gynogenesis than in conventional matings.

### Phenotypes Isolated and Complementation Analysis

A total of 77 candidate mutations have been isolated so far (Figure 2). These displayed phenotypes in a variety of developmental processes, and were classified into ten broad categories comprising those showing defects in the formation or acquisition of: eye, inner ear, and otolith; body axis; axial extension; pigmentation; head structures; gut; circulation; cardiovascular system; and motility. Images and/or movies and brief descriptions of these phenotypes can be viewed at http://www.nimr.mrc.ac.uk/devbiol/zimmerman.

Complementation analysis was performed within a few groups of similar mutant phenotypes (Table 4). Crosses were carried out among carriers of three mutations affecting otolith formation, three affecting jaw structure, two affecting axis length, and four affecting cardiovascular development. Two cardiovascular mutation carriers, *mlo^mh187^* and *mlo^mh19^,* failed to complement based on the observation of a highly specific paralysis and heart abnormality phenotype. In all other cases, crosses resulted in low incidence (<1%) of specific abnormalities, consistent with observed wild-type background phenotypic variation, suggesting that these independent alleles complemented each other. It should therefore be possible to isolate many additional alleles with this screen design.

### Representative Phenotypes

#### Axis extension phenotypes.

A subset of the mutations was selected for additional phenotypic analysis. Five lines produced embryos with relatively normal head and trunk structures that were defective in tail extension: *tansoku (tan), issunboushi (iss), david (dvd), kobito (kbt),* and *yodaa (yod).* We performed immunohistochemical analysis for laminin 1 expression on *tan, iss, kbt,* and *yod* mutant embryos, both to visualize axial structures and because three short-axis zebrafish mutations, *bashful, sleepy,* and *grumpy,* are caused by mutations in laminin subunits and result in severely depleted laminin 1 immunoreactivity [29,30]. At stage 41 the mutants *tan, iss, kbt,* and *yod* display relatively normal levels of laminin 1 staining, but evince distinct defects in somite structure (Figure 3). Laminin-stained *tan* embryos (Figure 3D) have relatively well-organized somites reduced in number relative to the wild type (Figure 3B), while *iss* (Figure 3H) and *kbt* embryos (unpublished data) manifest much greater disorganization of the intersomitic boundary. In *yod* embryos (Figure 3F) somite disorganization is limited to the trunk region, while somite width is reduced in the tail.

#### *mrs lot* embryos are defective in neural and cardiac function.

Another mutation, *mrs lot (mlo),* develops with grossly normal morphology (Figure 4A [wild-type] and 4B *[mlo]*) but fails to swim or to exhibit a tactile response when prodded with forceps. We stained *mlo* tadpoles with the HNK-1 antibody to visualize neuromuscular connectivity, and observed a disruption in motor neuron axon tracts. In wild-type tadpoles, these tracts travel from the neural tube down the intermyotomal cleft (white arrow, Figure 4C), while *mlo* axons wander away from the intermyotomal space (black arrow, Figure 4D). Muscle development is otherwise normal, as visualized by in situ hybridization with a cardiac actin probe (Figure 4E and 4F). While this neuromuscular connectivity deficit is consistent with the observed paralysis, we have not excluded the possibility of an upstream defect in neuronal excitability or metabolism.

#### *whitehart* affects hematopoiesis.

The *whitehart (wha)* mutation produces embryos with relatively normal heart function that lack circulating blood. Possible causes include vascular impairment or a deficiency in haematopoiesis. Whole-mount in situ hybridization for α-T4 globin shows that a small amount of blood appears ventrally in *wha* embryos (black arrows, Figure 5A and 5C), but it is difficult to quantitatively compare wild-type staining, which is distributed throughout the circulatory system. Comparison of α-globin in *wha* embryos (arrows, Figure 5C) with *muzak* (white arrows, Figure 5B), in which blood forms normally but remains localized to its ventral origin due to a defect in heart function, shows that *wha* haematopoiesis is profoundly affected and that impaired circulation is unlikely to be the underlying cause. A role for *wha* in haematopoiesis is also supported by microarray analysis discussed below.

#### *cyd vicious* results in neural crest and eye defects.

The *cyd vicious* phenotype (Figure 6) presents as superficially albino, paralyzed embryos with a faint dorsal stripe of pigmentation (arrow, Figure 6B). Feulgen-stained cross sections show pigmented cells in the lumen of the neural tube, consistent with a defect in neural crest migration (compare arrows, Figure 6C and 6D). Eye formation is also disrupted, with the formation of a lens-less hollow ball of neural retina (Figure 6F), again containing a small amount of pigmented tissue possibly related to prospective retinal pigmented epithelium, which is not neural crest derived. It remains to be determined whether this eye phenotype is intrinsic or an indirect result of defective crest-derived ocular mesenchyme interactions.

### Molecular Identification of Mutated Genes

Chemical mutagenesis is highly efficient at creating lesions, but associating a particular phenotype with a point mutation can be laborious in the absence of other molecular data. The availability of the X. tropicalis genomic sequence assembly, and the recent release of a meiotic map of SSR markers (http://tropmap.biology.uh.edu/map.html), greatly facilitate strategies for identification of candidate genes. We initially used a map-independent strategy, bulked segregant analysis of amplified fragment-length polymorphisms, to rapidly identify and clone markers linked to the *muzak* phenotype (unpublished data). Sequences from these markers were then placed on genomic sequence scaffolds 289, 567, and 158 (assembly V4.1), which were subsequently shown to map within a 7 cM interval on Linkage Group 1 of the independently derived SSR meiotic map. Additional mapped SSR markers are being used to refine the *muzak* interval prior to testing candidate open reading frames by mRNA or transgenic rescue and phenocopy by antisense morpholino oligonucleotide knockdown.

### A Pilot TILLING Screen and Estimation of Mutation Rates

In addition to our forward genetic screen, we tested the feasibility of a reverse genetic TILLING approach to obtain mutant phenotypes in known sequences. This also allowed us to simultaneously obtain a direct measurement of induced mutation rates. TILLING involves amplification of defined genomic sequences and identification of carriers of induced mutations in a panel of mutagenized animals (Figure 7). We have chosen to detect mutations by direct sequencing of amplified regions. Since the forward gynogenetic screen only makes use of F1 females, half of our mutagenized population was available for use. However, mutagenesis of mature sperm (G0) results in each strand of sperm DNA carrying a different constellation of mutations, leading to animals that are both heterozygous and mosaic at any induced loci, which interferes with direct sequence analysis in the F1 generation. Accordingly, in an initial feasibility study, testes from F1 males derived from the 10-mM ENU treatment were harvested, both to generate a frozen sperm library [31] for subsequent recovery of identified mutations and to outcross to produce a nonmosaic library of tadpole F2 genomic DNA to screen. This permitted a relatively quick screen of the library and measurement of mutation rate, but has the disadvantage that identified mutations must be recovered from mosaic F1 frozen sperm. With the intention of screening the spectrum of mutations present in the 63 F1 parents, we aimed to collect 24 F2 tadpoles per parent, and ultimately generated a library of 1,395 F2 tadpoles.

**Figure 8:**
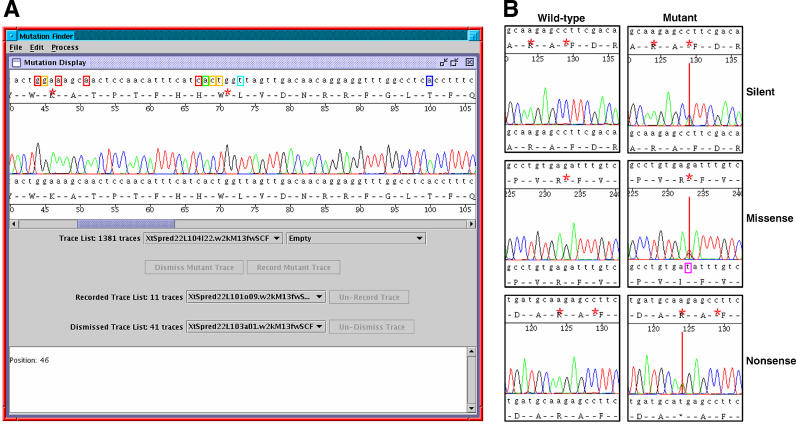
Mutation Detection All sequences generated by TILLING (see Figure 7) are examined and compared to a reference sequence by a Mutation Finder program. Any disparities with reference sequences are recorded for view in a Mutation Display window (A). Reference DNA and amino acid sequence is displayed above the trace and the TILLING trace below. Boxes around reference sequence nucleotides denote alterations in one or more TILLING traces; box color indicates number of traces altered. The asterisk above the reference amino acid sequence designates a position at which a mutation has been visually confirmed and recorded. Clicking on a box or asterisk will recover the trace(s) containing the change. Traces that are not confirmed are dismissed. All processed traces are accessible via the pull down trace lists. Examples of mutations are displayed for silent, missense, and nonsense alongside wild-type traces for comparison (B).

Genomic DNA was recovered from 5-d-old tadpoles, screened by nested PCR and direct sequencing of amplicons (Figure 7). To bias towards detection of nonsense mutations giving loss of function phenotypes, primers were designed to amplify 150–350 bp of amino-terminal exons. Forward sequence was generated for each of the 1,395 PCR products for each of the amplicons, and processed by a Mutation Finder program [32] that compares each trace to a reference sequence and identifies potential mutations. Data for each amplicon were then visually corroborated in a Mutation Display window (Figure 8A), and individual heterozygotes were confirmed if they display both a reduced height of a wild-type peak and no background noise throughout the trace. Mutations can be distinguished from the single nucleotide polymorphisms by analyzing the distribution of an identified base change within the mutagenized population, which was derived from a single pair of animals. If a base change was only seen within one group of 24 sibling tadpoles it is categorized as a mutation; alternatively, if it was observed throughout multiple families, it was classed as a single nucleotide polymorphism. In addition, single nucleotide polymorphisms are unlikely to be mosaic in the F1 generation, and are detected in ~50% of F2 progeny in a given family. Examples of mutations and their wild-type counterparts are illustrated in Figure 8B.

We employed this approach to measure the induced mutation rate for the 10 mM ENU–treated population. In a duplicate experiment screening six amplicons, we confirmed 27 base changes in approximately 1.4 MB of sequence (Table 5). Using these data, we estimate a mutation rate of one base change every 102,853 ± 41,030 bases. The variations observed in the rates for different genes are likely to be a consequence of the relatively small number of mutations detected thus far.

### Recovery of Mutations in Known Genes

Five nonsense mutations confirmed by duplicate TILLING experiments were selected for further analysis. Four of these mutations were observed in one of 24 siblings screened, but the fifth, in NFATC3 (nuclear factor of activated T cells cytoplasmic 3) [33], was detected in five siblings of 24. The cognate mosaic F1 frozen sperm samples were used to carry out in vitro fertilization. One mutation was unrecoverable due to poor frozen sperm quality. Three mutations were not recovered despite screening more than 90 embryos in two cases, which is consistent with a high degree of mosaicism in the F1. However, the NFATC3 nonsense mutation was identified in three of 16 tadpoles, a similar frequency to that observed in the screen. As this mutation deletes the carboxy-terminal two-thirds of the protein, it is likely to result in a loss of function phenotype when bred to homozygosity. Identified carrier frogs are currently being raised for phenotypic analysis.

### Microarray Studies with Mutant Embryos

Anticipating that a large number of hybridization experiments would be required for the routine analysis of X. tropicalis gene expression, we decided to generate our own microarrays using long synthetic oligonucleotides as probes. In the choice of probe sets we took several factors into consideration. First, we decided that the most informative set of probes would represent as large a set of well-characterized genes as possible; hence, we have included all of the genes available on the *Xenopus* Molecular Marker Resource (http://www.xenbase.org/xmmr) for which an X. tropicalis ortholog clearly exists. Second, we incorporated genes whose expression pattern has been described and which are available on public *Xenopus* and zebrafish databases. Thus, we have included a large number of X. tropicalis genes that are orthologous to the X. laevis genes described on the Axeldb site (http://www.dkfz-heidelberg.de/molecular_embryology/axeldb.htm) [34–36]. We also included ~500 genes whose expression is described on the Zebrafish ZFIN database (http://zfin.org). Finally, we were informed by a large and more-comprehensive developmental study done with zebrafish, in which Affymetrix microarrays were used in a developmental time course covering the first 5 d of zebrafish development (Martina Konantz and Robert Geisler, personal communication; data available through Microarray Express [http://www.ebi.ac.uk/arrayexpress], experiment E-TABM-33). From this set of genes, we selected a subset of genes whose expression was found to change in a statistically significant manner by at least 2-fold. We then identified as many X. tropicalis orthologs as we could by comparing the zebrafish mRNA sequence to X. tropicalis full-length cDNAs, and Ensembl (http://www.ensembl.org/Xenopus_tropicalis/index.html) transcripts identified as known genes (with strong supporting evidence). This collection of X. tropicalis sequences was used to design 65-mer oligonucleotide probes for our microarray, using the complete Joint Genome Institute (Walnut Creek, California, United States) set of 27,916 set of predicted genes (September 2005) as the comparison genome for uniqueness. Currently, the oligonucleotide set includes probes for 2,350 X. tropicalis genes. We had slides printed (with oligos in duplicate) and quality-controlled by the Sanger Institute microarray facility, and have now performed several pilot experiments using the arrays.

To assess the efficacy of microarray expression profiling for analysis of mutant phenotypes, we compared gene expression at stages 37–38 in an X. tropicalis mutation identified in our forward screen, *wha,* to stage-matched wild-type siblings. Comprehensive data are available on ArrayExpress (experiment accession number E-MEXP-691[http://www.ebi.ac.uk/arrayexpress]). We found that 216 of the 2,350 genes represented in our microarray showed statistically significant >2-fold changes in level of expression in *wha* embryos. Consistent with the “bloodless” phenotype of this mutation as detected by in situ hybridization (Figure 5), we find that hemoglobin and heme biosynthesis genes are among the most highly downregulated genes detected in microarray experiments (Table 6). We also found significant downregulation of the BMP4-responsive gene *vent-1,* which is involved in specification of ventral fates in early embryos [37,38]. This observation may indicate a failure of aspects of ventral specification in *wha* mutants; alternatively, *vent-1* may be more directly involved in blood or vascular development. Among the genes found to be upregulated in *wha* mutants is *cytokeratin-18* (*krt18;* Table 7), which encodes an intermediate filament protein normally expressed in epithelia. The design of our microarray, which is biased toward well-characterized genes, allows us access to a variety of whole-mount in situ hybridization expression pattern databases. In this case the zebrafish ZFIN database has a rich set of images detailing the *krt18* expression. At the corresponding stage of the *wha* mutants analyzed here, this gene is strongly expressed in the zebrafish cardiovascular system. Microarray analysis thus gives a valuable global snapshot of gene expression changes in this mutant relative to the wild type, providing insights for further characterization of changes in specific processes.

## Discussion

This report presents the first forward and reverse genetic screens for chemically induced mutations in *X. tropicalis.* We have isolated a diverse set of heritable phenotypic abnormalities in organogenesis and differentiation, and also successfully identified frogs carrying mutations in known genes for use in future studies of specific gene functions. Some of the phenotypes isolated in our forward screen resemble those already uncovered in other model systems, validating our screen design. Others, such as the *cyd vicious* phenotype, do not appear to resemble known mutations, and confirm that X. tropicalis forward genetics can be a highly effective tool for discovery of novel gene functions. While this pilot screen clearly demonstrates the feasibility of the approaches we have taken, there are a number of factors to consider for future screens.

### Gynogenetic Forward Screen Limitations

In our screen strategy, in vitro mutagenesis followed by gynogenesis and morphological inspection was employed to perform rapid surveillance of postneurulation phenotypes. While this approach permits screening only one life cycle after mutagenesis and greatly reduces colony requirements, several types of bias are introduced. First, highly penetrant phenotypes in early development will not be isolated in this pilot screen. Embryos with these types of defects can be difficult to identify on the complex background of nonspecific gastrulation defects associated with early pressure gynogenesis and incomplete rescue from the haploid state. The overlooked gene functions may include many housekeeping genes required for cell survival, as well as a number of tissue-specific zygotic genes involved in early tissue movements and embryogenesis. Replacement of early pressure with early cold shock to effect gynogenesis reduces this background noise to a degree, and may permit isolation of earlier phenotypes. Second, gynogenesis by suppression of polar body formation is biased towards recovery of centromerically linked loci due to meiotic recombination [5,20,27,28], so effectively only this subset of the genome is being screened. This bias comes with the significant advantage that mapping studies can focus on these centromeric regions. In some cases, we have observed additional phenotypes segregating in Mendelian ratios in the F3 progeny of sibling crosses, which were not observed in gynogenetic embryos, consistent with recovery of more distal alleles. Third, in vitro mutagenesis of mature sperm results in a mosaic F1 generation, further reducing the frequency with which recessive gynogenetic phenotypes may be observed. We chose an arbitrary number of 20 viable gynogenetic F2 neurulae as a practical threshold at which to consider an F1 genome screened; examination of larger numbers of embryos is likely to result in the identification of additional phenotypes. Likewise, germline mosaicism can also interfere with recovery of detected phenotypes, so we have designated as “provisional” (color-coded green in Tables 2 and 3, and Figure 2) alleles that have not yet been shown to be heritable. However, it should be noted that 100% (29/29) of those phenotypes which have been observed twice in the gynogenetic progeny of F1 founders have been shown to be heritable thus far. As a practical point, this suggests that it should be feasible to perform preliminary characterization of a large number of provisional alleles, which can be maintained long-term as individual F1 founder frogs. Limited colony resources can then be dedicated to breeding specific phenotypes of interest, without undue risk that many will not be heritable. Replacing gynogenetic screens with a conventional three-generation screen design is unwieldy in combination with in vitro mutagenesis, as mosaicism in the F1 animals greatly reduced the frequency with which nonmosaic F2 carriers will be randomly paired in matings to produce F3 embryos to screen. Finally, morphological inspection is a relatively superficial approach, and additional phenotypes might be recovered by use of in situ hybridization or mutagenesis of transgenic multireporter strains [13] to detect more subtle variations in gene expression.

### Structure of Induced Mutations

We initially chose to pursue in vitro chemical mutagenesis of postmeiotic sperm, rather than in vivo spermatogonial treatment, for speed of analysis and efficiency of mutation induction. One drawback to this approach is that the F1 animals generated by in vitro fertilization with mutagenized sperm are mosaic, and hence phenotypes in their progeny may not appear in Mendelian ratios. As our forward genetic screen was based on gynogenesis, which precludes the production of Mendelian ratios, this was not a significant problem. In addition, mutations induced by postmeiotic sperm mutagenesis in other vertebrates include not only point mutations [39,40] but also deletions and chromosomal rearrangements [41–43]. Deletions can be highly useful genetic resources for functional and mapping studies, but as yet neither deletions nor translocations have been identified among our induced mutations. The majority of mutations that have been subjected to F2 sibling intercrosses in our study have produced Mendelian phenotypic ratios in F3 progeny, which are not indicative of gross chromosomal defects [28]. While it is possible that multigene deletions are induced in our protocol, these may result in a higher frequency of early lethal phenotypes, which we have discarded. It has also been proposed that subtle differences in mutagenesis conditions may result in significant differences in the kinds of lesions produced [41]. What is clear is that the sequence of a large number of specific amplicons in our mutagenized population indicates a high frequency of induced point mutations. While direct sequencing will not detect deletions or rearrangements, we conclude that single base changes are induced highly efficiently in our protocol. Mapping and cloning studies will ultimately be required to confirm whether point mutations or deletions are responsible for the majority of the phenotypes observed in the forward screen.

Direct sequencing of genomic PCR products also confirms a varying degree of mosaicism in the F1 generation, as demonstrated by the observation of one mutation at a frequency of ~1/5 and others at 1/24 or less. In the first case, the observed ratio is in line with the expected result of in vitro ENU mutagenesis of mature sperm. If a DNA adduct forms on one strand of sperm DNA, that lesioned strand might be expected to be distributed to one of two cells at first cleavage, resulting in a 50% mosaic heterozygous embryo, such that on average 25% of the germline might carry a specific lesion. The majority of the phenotypes obtained in the forward screen are likely to be of this less-mosaic category. Two explanations are likely for the higher observed level of mosaicism in some of the other families. One is that there may be a degree of selection in the F1 spermatogonia against individual germ cells carrying particular combinations of mutations. The second possibility is that repair of modified bases carried by the mutagenized sperm may be delayed for a number of cell divisions, resulting in a significantly higher degree of mosaicism, and a lower frequency of specific F1 carriers. Consistent with this notion, it is not until later stages of development that cells carrying damaged DNA are recognized and eliminated by apoptosis [44,45].

Several observations suggest that the majority of these mutations are induced, rather than already present in the strain of frogs used. Naturally occurring recessive alleles have been found in an inbred *N* (Nigerian) strain [46], and a number have been recovered from outbred wild-caught X. tropicalis [21]. One of these naturally occurring mutations, *grinch,* has been identified in our *N* strain stock and confirmed in complementation tests (Tim Grammer and Richard Harland, personal communication). Since the stocks used for mutagenesis are the grandchildren of a single pair of animals, a maximum of four alleles preexisted at each locus in the population. We would therefore expect background mutations to be recovered several times in a pilot of this size, as indeed we have seen with *grinch.* While we have only shown complementation among a limited number of mutations, the majority of the remainder are phenotypically distinct, consistent with induced mutations in a number of different genes. In addition, gynogenesis of nonmosaic F2 animals can also provide an indication of whether loci are distinct, since the frequency with which recessive phenotypes are uncovered is inversely dependent on meiotic recombination rate, a function of the gene–centromere distance [47]. Mutations that are recovered with different frequencies in the gynogenetic progeny of nonmosaic females are likely to be distinct loci. Finally, the appearance of mosaicism in the F1 generation, which is expected in the products of in vitro mutagenesis of mature sperm, also supports the induced nature of the mutations. This mosaicism is evident in Table 3, in which the preponderance of mutant phenotypes are observed at a lower frequency in the gynogenetic progeny of F1 females compared with those of (nonmosaic) F2 females. Background recessive mutations would be expected to be nonmosaic in both the F1 and F2 generations, and the ratio recovered in the gynogenetic progeny of carrier females would not be expected to change.

### Reverse Screen

This TILLING screen demonstrates the feasibility of identifying and recovering carriers of mutations in known genes. Our pilot scheme, designed for speed, was somewhat hampered by dependence on recovering carriers among the progeny of variably mosaic F1 animals. Efficiency in future screens can be increased by sequencing exons from an adult nonmosaic F2 mutagenized population, among whose progeny carriers can be expected to be recovered in predictable Mendelian ratios. Accordingly, we are in the process of generating a library of F2 DNA and germline stocks (maintained both as frozen sperm and living frogs), which will greatly enhance recovery of identified mutations. While maintaining a living library is more space- and labor-intensive, fertilization yield from an individual X. tropicalis' frozen testes is typically limited to about 500 embryos, and sperm viability after freezing can vary. X. tropicalis females are fecund (producing up to 9,000 eggs per ovulation and capable of breeding six times per year [14]), long-lived, and are likely to be fertile for more than 10 y (in contrast to zebrafish or mice, with a typical fertility span of less than 2 y). As each individual X. tropicalis may harbor a number of different mutations, it may be very useful to be able to perform multiple screens over a decade. Resequencing a large number of X. tropicalis exons validates the quality of the genome assembly as well as the annotation used to identify intron/exon boundaries for primer design. These data also provide a useful estimation of the induced mutation rate, which was found to be about double that calculated in zebrafish and rat TILLING screens following spermatogonial mutagenesis [10,48].

### Microarray Analysis

Microarrays are the most efficient means to quickly obtain expression information for thousands of genes. We have utilized the exceptional sequence resources now available for X. tropicalis to construct custom microarrays for a first-pass analysis of phenotypes identified in forward and reverse screens. By choosing well-described genes with known expression patterns and functions, our array design seeks to provide a snapshot description of phenotypes in order to suggest further strategies for characterization. Comparison of gene expression patterns in the *wha* mutation relative to wild-type embryos confirms that we can observe specific changes consistent with the observed morphological phenotype, as well as obtain new information suggesting fresh avenues for investigation. The combination of these molecular phenotyping tools with the mutagenesis procedures and genetic screens described here helps to establish an infrastructure for obtaining and analyzing chemically induced phenotypes in the emerging model vertebrate Xenopus tropicalis.

## Materials and Methods

### Frog strains.

Mutagenesis and in vitro fertilization were performed with *N* (Nigerian) strain animals (kind gift of Enrique Amaya, Cambridge University, United Kingdom); polymorphic crosses were generated using the *IC* (Ivory Coast) strain (kind gift of Robert Grainger, University of Virginia, Charlottesville, Virginia, United States).

### Chemical mutagenesis.

A 100-mM ENU stock (*N*-nitroso-*N*-ethylurea; Sigma N3385, isopac; Sigma, St. Louis, Missouri, United States) was prepared in 5 mM MES (2-[N-morpholino] ethanesulfonic acid [pH 6.0]; Sigma M-3671). Typically, eight to ten testes were dissected from wild-type *N* strain males, dissociated with an Eppendorf pestle in 1 ml Leibovitz L15 medium (catalog no. 31415–029; GIBCO, San Diego, California, United States) and incubated in L15 + 3 mM MES (pH 6.2) ± ENU for 1 h at 18 °C. Treated, dissociated testes were then washed twice with L15 and used to fertilize eggs from wild-type *N* strain females to generate the F1 candidate carrier mutagenized populations and controls.

### Gynogenesis.

Sperm preparation and gynogenesis were essentially as previously described [21] with minor modifications: testes were kept in L15 + 10% calf serum at 14 °C, dissociated, placed on a glass petri dish and exposed to one UV treatment of 50,000 μJ/cm^2^ in a Stratalinker 2400 (Stratagene, La Jolla, California, United States), except for an nonirradiated aliquot for diploid (outcrossed sibling) controls. Five min after fertilization with irradiated sperm, ~80% of the haploid embryos were treated with 3,000–3,500 psi pressure for 6 min to suppress second polar body formation and produce gynogenetic diploid embryos. The remaining embryos were allowed to develop as haploids. Alternatively, for some of the later analyses polar body suppression to form gynogenetic diploids was effected by coldshock rather than pressure (personal communication; for protocol contact Rob Grainger, University of Virginia).

### Screening.

Epigenetic effects and incomplete rescue of haploids can produce a high background of early developmental defects in gynogenetic diploid *X. tropicalis.* To circumvent this developmental noise, embryos were allowed to develop through neurula stages then sorted and those with early developmental defects discarded. Phenotypically normal neurulae from gynogenetic diploid and haploid dishes were then allowed to continue to develop at 25 °C and compared to outcrossed sibling controls at 2, 3, and 4 d after fertilization. Two-d-old embryos (stages ~35–37) were assessed for qualitative differences from wild-type out-crossed siblings in general morphology, axis elongation, tactile responsiveness/motility, heart rate and rhythm, blood circulation, eye morphology and pigmentation, melanophore differentiation, and migration and somite structure. Three-d-old embryos (stage ~42) were screened for the above as well as motility, gut folding, pigmentation, and appearance of otoliths. At 4 d (stage ~45) the embryos were also scored for shape and size of otoliths, otic vesicle, rhombomere morphology, gut looping, chirality of intestine and heart, and shape of head and mouthparts.

### Histological analysis.

For histological analysis, embryos were processed to generate 7-μm paraffin sections, and stained with Feulgen as previously described [49].

### Immunohistochemistry.

Immunohistochemistry was carried out as previously described [50], with a 1:500 dilution of anti-laminin (Sigma L9393) in PBST + 20% nonimmune serum, followed by 1:400 HRP-conjugated anti-rabbit secondary antibody (111-035-114; Jackson ImmunoResearch, West Grove, Pennsylvania, United States); or a 1:200 monoclonal anti-HNK1/NCAM antibody (Sigma C-0678), followed by HRP-conjugated anti-mouse IgM (Sigma A8786) at 1:500.

### Whole-mount in situ hybridization.

In situ hybridization was carried out as previously described [51]. X. laevis probes (cardiac actin [52], kind gift from Dr. Tim Mohun, and αT4 globin probe [53], kind gift from Prof. Roger Patient) were both linearized with EcoRI and transcribed with SP6 polymerase.

### Genomic DNA library.

Five-d-old F2 tadpoles were euthanized by immersion in 3.8 mM ethyl 3-aminobenzoate methanesulfonate. Tadpoles were placed into individual wells in deep 96-well plates and frozen on dry ice. Genomic DNA was prepared from frozen tadpoles essentially as described by Weinholds et al. [10], with an overnight incubation in a 55 °C shaking oven to promote tadpole lysis. The working stocks were aliquoted from the master 96-well plates into deep 384-well plates using a Quadra96 robot (Tomtec, Hamden, Connecticut, United States). The genomic DNA library master plates were kept at −20 °C and working stocks were stored at 4 °C.

### Primer design and PCR.

Genes of interest were identified and X. laevis or X. tropicalis sequences blasted against the X. tropicalis genome assembly in Ensembl to identify predicted gene structure (http://www.ensembl.org/Xenopus_tropicalis). The Ensembl gene ID or Fasta formatted sequence (when no Ensembl gene was found) was then used to set up a project in LIMSTILL (Laboratory Information Management Systems for the identification of mutations by sequencing and TILLING; http://limstill.niob.knaw.nl); this contains a primer3 [54]–based program that can design nested primers to amplify exons of interest (Table 8). Nested primers for the second PCR were M13 coupled, resulting in 150–450 bp amplicons. The primers were tested using a gradient PCR to find the optimal annealing temperature for the first PCR. The gradient PCR mixture contained 4 μl genomic DNA, 2 μM f1 and 2 μM r1 primer, 200 μM dNTPs, 0.4 U Taq DNA polymerase, 25 mM Tricine, 85 mM NH_4_ Acetate (pH 8.7), 2 mM MgCl_2_, 8.0% glycerol (m/v), and 1.6% DMSO (m/v), in a final volume of 20 μl. The reactions were cycled on DNA Engine Tetrads (MJ Research, Waltham, Massachusetts, United States) using a gradient PCR program as follows 94 °C for 3 min, (94 °C for 45 s, 50–64 °C for 45 s, 72 °C for 1.5 min) × 35 cycles, 72 °C for 10 min, and 10 °C forever.

**Table 8:**
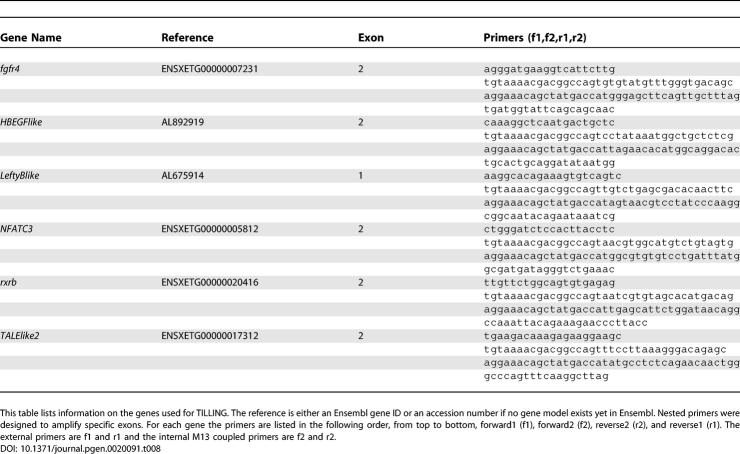
Gene and Primer Data for TILLING

Nested PCR reactions for the screen were set up in 4 × 384–well plates with genomic DNA from the library as template. The PCR1 reaction was set up as above except the final volume was 10 μl. Genomic DNA (2 μl) was aliquoted from working stocks into 384-well plates for PCR using a Quadra384 robot (Tomtec). PCR mixture (8 μl) was dispensed into the 384-well plates using a Multidrop Micro (Thermo, Waltham, Massachusetts, United States). The PCR program was as above except the gradient annealing temperature was replaced with the optimal empirically determined temperature for each specific PCR1. The PCR2 mixture was different from PCR1 as it contained no genomic DNA but was still at a final volume of 10 μl/well. The aliquoted PCR2 mixture was inoculated twice from PCR1 using a 384-pin hedgehog (Genetix, Cambridge, Massachusetts, United States). The reactions were then cycled as above but with a 58 °C annealing temperature.

The PCR products were cleaned for sequencing by addition of 0.5 U shrimp alkaline phosphatase, 2 U exonuclease I, 0.25 M Tris-HCl (pH 8.5), and 25 mM MgCl_2_ in a final volume of 5 μl and incubation at 37 °C for 1 h, followed by inactivation at 80 °C for 15 min. PicoGreen (Invitrogen, Carlsbad, California, United States) was used to quantify the PCRs and they were then diluted to 10–15 ng/μl for sequencing.

### Sequencing and sequence analysis.

Samples were sequenced using M13 forward primer and a 1/64 dilution of BigDye v3.1 terminator (Applied Biosystems [ABI], Foster City, California, United States) standard recipe. The reactions were cycled on DNA Engine Tetrads (MJ Research) at 96 °C for 30 s (92 °C for 5 s, 50 °C for 5 s, 60 °C for 2 min) × 45 cycles, 10 °C forever. The reactions were precipitated in 68% ethanol and 40 mM sodium acetate, spun at 4,000 rpm at 4 °C for 30 min, washed in 80% ethanol, and spun for a further 5 min. After drying the reactions were resuspended in 0.1 mM EDTA and loaded onto an ABI 3730 DNA sequencer, with a 36-cm array.

### Mutation identification.

Sequence data were analyzed using Java-based programs written by DS as previously described [32]. These are available upon request.

### Microarray production.

X. tropicalis sequences were selected from Xenbase (http://www.xenbase.org), from public full-length cDNA or Ensembl transcript sequences based on homology to Danio rerio or X. laevis marker genes. PolyA tails were trimmed using trimest (http://www.emboss.org), reduced to the most 3′ 500 bp using a custom script, and repeats were soft-masked using RepeatMasker (http://www.repeatmasker.org). Arrayoligoselector (http://arrayoligosel.sourceforge.net) [55] was used to design 65-mer oligonucleotides with ~50% GC content that were unique when compared to all predicted transcripts from JGI version 4.1 (http://genome.jgi-psf.org/Xentr4/Xentr4.home.html). Oligonucleotides with 5′ amino-link were obtained from Illumina, spotted in duplicate, and processed by the Sanger Microarray Facility on Codelink activated slides (GE Healthcare, Milwaukee, Wisconsin, United States) according to the manufacturer's instructions. Each transcript is represented by one oligonucleotide spotted twice on each array.

### RNA isolation, labeling, and hybridization.

Two batches each of 40 *wha* mutant and 40 wild-type sibling embryos were sorted by morphological criteria at stages 37–38, snap-frozen, and stored at −80 °C until use. Embryos were lysed in Trizol (Invitrogen) and processed in Phase Lock Gel tubes (Eppendorf, Hamburg, Germany) according to the manufacturer's instructions. The dissolved RNA was further purified by lithium chloride precipitation [56]. Total RNA was quantified on a NanoDrop spectrophotometer (NanoDrop, Wilmington, Delaware, United States), and 40 μg of each were used in Amino Allyl cDNA labeling kit reactions (Ambion, Austin, Texas, United States). The resulting modified cDNA samples were split in half, one labeled with monoreactive Cy3 and the other with monoreactive Cy5 (GE Healthcare) to provide dye-swapped replicas for each sample. The dye-labeled cDNAs were purified using Qiaquick columns (Qiagen, Valencia, California, United States), and paired labeled probes (wild-type versus *wha* siblings) were mixed with 8 μg of Cot1 (Invitrogen), 4 μg polyA (Sigma), 250 μg sheared salmon sperm (Ambion), and ethanol precipitated. The pellets were washed, dried, and resuspended in 10 μl water, heated to 70 °C for 5 min; then, 50 μl hybridization buffer (50% Formamide, 5 × SSC, 0.1% SDS, 0.1 mg/ml BSA) was added, mixed, and heated to 70 °C for 10 min. The sample was cooled to room temperature, and any particulates pelleted. A portion of the mixture (55 μl) was added to each microarray using a 25 × 60–mm coverslip. Hybridizations were carried for 16 h at 42 °C in a SlideBooster (Advalytix, Brunnthal, Germany) with the following settings: PWMRatio = 0.3 and MixPower = 21.

### Posthybridization analysis.

Slides were washed three times in 0.1 × SSC and 0.1% SDS for 15 min, and three times for 5 min in 0.1 × SSC. All washes were at room temperature with gentle agitation. Slides were spun dry and scanned at 10-μm resolution on a ScanArray HT and analyzed using ScanArrayExpress (PerkinElmer, Wellesley, California, United States). Image analysis results were passed from ScanArrayExpress in GenePix Results format into GeneSpring 7.0 (Agilent, Palo Alto, California, United States) and locally weighted scatterplot smoothing normalized. Genes were identified as significant if they fulfilled these three criteria: marked as present in at least half the experiments, changed expression by at least 1.5-fold, had a *t* test *p* ≤ 0.05.

## Supporting Information

### Accession Numbers

The GenBank (http://www.ncbi.nlm.nih.gov/Genbank) accession numbers for the products discussed in this paper are cardiac actin (X04669) and α-T4 globin (X02797).

## **Abbreviations:**

*iss*: *issunboushi*
*kbt*: *kobito*
*mlo*: *mrs lot*
NFATC3: nuclear factor of activated T cells cytoplasmic 3
SSR: simple sequence repeat
*tan*: *tansoku*
TILLING: targeting induced local lesions in genomes
*wha*: *whitehart*
*yod*: *yodaa*
